# Nuclear DICKKOPF-1 as a biomarker of chemoresistance and poor clinical outcome in colorectal cancer

**DOI:** 10.18632/oncotarget.3464

**Published:** 2015-02-28

**Authors:** Óscar Aguilera, José Manuel González-Sancho, Sandra Zazo, Raúl Rincón, Agustín F. Fernández, Olga Tapia, Francesc Canals, Beatriz Morte, Vincenzo Calvanese, José L. Orgaz, Núria Niell, Susana Aguilar, José M. Freije, Osvaldo Graña, David G. Pisano, Aurea Borrero, Javier Martínez-Useros, Benilde Jiménez, Mario F. Fraga, Jesús García-Foncillas, Carlos López-Otín, Miguel Lafarga, Federico Rojo, Alberto Muñoz

**Affiliations:** ^1^ Instituto de Investigaciones Biomédicas “Alberto Sols”, Consejo Superior de Investigaciones Científicas (CSIC), Universidad Autónoma de Madrid, Madrid, Spain; ^2^ Instituto de Investigación Sanitaria-Fundación Jiménez Díaz, Madrid, Spain; ^3^ Cancer Epigenetics Laboratory, Instituto Universitario de Oncología del Principado de Asturias (IUOPA-HUCA), Universidad de Oviedo, Oviedo, Spain; ^4^ Departamento de Anatomía y Biología Celular, Universidad de Cantabria-IFIMAV, Santander, Spain; ^5^ Proteomics Laboratory, Vall d'Hebron Institute of Oncology (VHIO), Vall d'Hebron University Hospital, Barcelona, Spain; ^6^ Centro de Investigación Biomédica en Red de Enfermedades Raras (CIBERER), Madrid, Spain; ^7^ Catalan Institute of Oncology-IDIBELL, L'Hospitalet de Llobregat, Barcelona, Spain; ^8^ Departamento de Bioquímica y Biología Molecular, Facultad de Medicina, Instituto Universitario de Oncología, Universidad de Oviedo, Oviedo, Spain; ^9^ Bioinformatics Unit and Structural Biology and Biocomputing Programme, Spanish National Cancer Research Centre, Madrid, Spain; ^10^ Department of Immunology and Oncology, Centro Nacional de Biotecnología (CSIC), Madrid, Spain; ^11^ Present address: The Scripps Research Institute, La Jolla, CA, USA; ^12^ Present address: Randall Division of Cell and Molecular Biophysics, King's College London, United Kingdom; ^13^ Present address: Department of Molecular, Cell and Developmental Biology, University of California Los Angeles, CA, USA

**Keywords:** Colorectal cancer, Dickkopf-1, chemoresistance, ALDH1A1, biomarker

## Abstract

Sporadic colorectal cancer (CRC) insurgence and progression depend on the activation of Wnt/β-catenin signaling. Dickkopf (DKK)-1 is an extracellular inhibitor of Wnt/β-catenin signaling that also has undefined β-catenin-independent actions. Here we report for the first time that a proportion of DKK-1 locates within the nucleus of healthy small intestine and colon mucosa, and of CRC cells at specific chromatin sites of active transcription. Moreover, we show that DKK-1 regulates several cancer-related genes including the cancer stem cell marker aldehyde dehydrogenase 1A1 (*ALDH1A1)* and Ral-binding protein 1-associated Eps domain-containing 2 (*REPS2),* which are involved in detoxification of chemotherapeutic agents. Nuclear DKK-1 expression is lost along CRC progression; however, it remains high in a subset (15%) of CRC patients (n = 699) and associates with decreased progression-free survival (PFS) after chemotherapy administration and overall survival (OS) [adjusted HR, 1.65; 95% confidence interval (CI), 1.23-2.21; *P* = 0.002)]. Overexpression of *ALDH1A1* and *REPS2* associates with nuclear DKK-1 expression in tumors and correlates with decreased OS (*P* = 0.001 and 0.014) and PFS. In summary, our findings demonstrate a novel location of DKK-1 within the cell nucleus and support a role of nuclear DKK-1 as a predictive biomarker of chemoresistance in colorectal cancer.

## INTRODUCTION

Colorectal cancer (CRC) is an important contributor to cancer mortality and morbidity [1]. Despite advances in therapy for CRC patients, approximately 20-45% of those who undergo curative resection subsequently develop tumor recurrence or distant metastasis [2]. Adjuvant chemotherapy has demonstrated survival benefits in patients with advanced-stage CRC, but the lack of patient selection leads to unnecessary overtreatment and financial cost [3]. To facilitate individually tailored treatment for CRC, predictive biomarkers are needed to identify patients who are at high risk for recurrence and/or poor prognosis.

Abnormal activation of the Wnt/β-catenin pathway due to mutation of *APC*, *CTNNB1*/β-catenin or *AXIN* genes is the initial event and a driving force of colorectal tumorigenesis [4]. Dickkopf (DKK)-1 is one of the four members of a family of secreted extracellular Wnt inhibitors that block signaling from plasma membrane Wnt-receptor complexes [5, 6]. Paradoxically, however, DKK-1 modulates proliferation and survival of cancer cells in which the Wnt/β-catenin pathway is constitutively activated by mutations in genes encoding intracellular pathway components, or independently of β-catenin transcriptional activity [7, 8]. Overall, available data indicate the existence of uncharacterized actions of DKK-1 that are independent of the inhibition of Wnt signaling at plasma membrane.

In colon cancer cells, DKK-1 inhibits proliferation both *in vitro* and in immunodeficient mice [8] and its expression is linked to an E-cadherin-dependent adhesive phenotype [9], supporting a tumor suppressor role for this protein. Moreover, several studies indicate that DKK-1 expression is downregulated along colorectal adenoma-carcinoma transition and at late CRC stages but the clinical consequences are unknown [8, 10]. By contrast, a recent study indicates that preoperative serum levels of DKK-1 protein seem to be a predictive marker of tumor invasion and relapse in stage II-III colon cancer [11]. DKK-1 is also downregulated in chronic lymphocytic leukemia and papillary thyroid cancer [12, 13]. These potential protective effects contrast with the overexpression of DKK-1 in several types of cancer associated with a poor prognosis [14-20], and make the role of DKK-1 as tumor suppressor or metastasis promoter a matter of debate [21].

In this study, we aimed to decipher the role of DKK-1 in CRC by investigating its expression in cultured human colon carcinoma cells and in normal small intestine and colon mucosa and colorectal cancer, and by assessing the relation of DKK-1 expression with the clinical outcome and the benefit to systemic therapy in CRC patients.

## RESULTS

### DKK-1 protein partially locates within the cell nucleus

**Figure 1:**
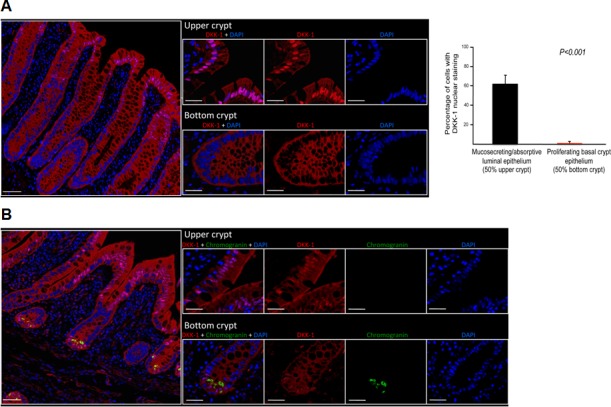
DKK-1 is present within the nucleus of differentiated human intestinal cells A, left, expression of DKK-1 in human colon crypts by immunofluorescence showing nuclear location in enterocytes and goblet cells at the tip of crypts, which is absent in proliferative and stem cells at the bottom. Statistical differences in nuclear DKK-1 expression were observed between upper and lower sections of the crypts (*P* = 10^−20^); right, data correspond to quantification of 320 randomly distributed crypts from 16 healthy individuals. B, strong nuclear and cytoplasmic DKK-1 expression in small intestine epithelial cells at the villus tips and in chromogranin-expressing enteroendocrine cells at the crypt bottoms. In both panels, scale bars: left images, 100 μm; right images, 20 μm. Nuclei were stained with DAPI.

First, we analyzed the expression of DKK-1 protein in human intestine mucosa using immunofluorescence multispectral microscopy of healthy individuals. Unexpectedly, we found nuclear staining in a high proportion (62.8%) of differentiated cells (i.e. enterocytes and mucosecretory goblet cells) located at the epithelium of colon (Fig. 1A) and small intestine (Fig. 1B) crypts. In the latter, nuclear DKK-1 expression was also detected in enteroendocrine cells at the bottom of the crypts, as revealed by co-expression of chromogranin A (Fig. 1B). Cytoplasmic staining was diffusely present in small intestine enterocytes and enteroendocrine cells (Fig. 1B). By contrast, stem cells at the bottom of the crypts and proliferating undifferentiated cells in the basal epithelia contained cytoplasmic DKK-1 but lacked nuclear DKK-1 in both colon and small intestine (Figs. 1A and B).

**Figure 2:**
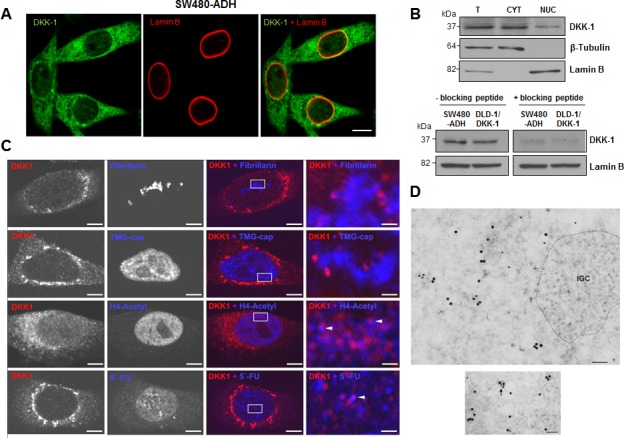
DKK-1 partially locates in the cell nucleus at sites of active transcription A, immunofluorescence and confocal microscopy images showing DKK-1 expression in the cytoplasm and nucleus of SW480-ADH colon cancer cells. Scale bar, 10 μm. B, upper, expression of DKK-1 protein in total (T), cytoplasmic (CYT) and nuclear (NUC; 10-fold concentrated) fractions of SW480-ADH cells by Western blot analysis. Lamin B and β-tubulin were used as nuclear and cytoplasmic markers, respectively. Lower, Western blot analysis of DKK-1 protein in nuclear extracts of SW480-ADH and DLD-1/DKK-1 cells using a DKK-1 antibody pre-incubated (overnight, 4°C) with a specific blocking peptide. C, lack of immunofluorescence co-staining with fibrillarin and TMG-cap shows that DKK-1 locates outside but close to nucleoli and nuclear speckles in SW480-ADH cells. By contrast, partial colocation with acetylated Histone H4 and 5′-FU (arrowheads) indicates that DKK-1 resides at active transcription sites. Scale bars: three left columns, 5 μm; right column, 0.8 μm. D, immunoelectron microscopy analysis using primary mouse anti-BrdU to detect 5′-FU incorporation into nascent RNA and rabbit anti-DKK-1 antibodies, and secondary goat anti-rabbit or anti-mouse IgG coupled to 15 nm or 10 nm gold particles, respectively. Upper, 5′-FU incorporation sites and DKK-1 appear distributed in euchromatin regions while they are absent in interchromatin granule clusters (IGC, dotted area), which are transcription-free nuclear domains. Lower, nascent RNA and DKK-1 protein colocate in euchromatin (arrows). Scale bars: 100 nm.

Next, we studied the expression of DKK-1 in human colon carcinoma cells both *in vitro* and in a large cohort of metastatic CRC patients. DKK-1 was found partially located within the nucleus of SW480-ADH CRC cells, as shown by punctate immunofluorescence staining (Fig. 2A) and confirmed and quantified by Western blot analysis of cellular fractions (Fig. 2B). Nuclear DKK-1 accounted for 7% of total protein. We also detected nuclear DKK-1 via proteomic analysis (Supplementary Fig. S1) and by transfecting DLD-1 CRC cells lacking endogenous expression with a *DKK-*1 gene (DLD-1/DKK-1 cells) (Supplementary Fig. S2).

To identify the precise intranuclear location of DKK-1, we performed double immunofluorescence assays. DKK-1 did not colocalize with either fibrillarin or 2,2,7-trimethylguanosine cap (TMG-cap), indicating that it is absent from the nucleolus and nuclear speckles, respectively (Fig. 2C). Sporadic colocation of DKK-1 with acetylated histone H4 indicated its presence in transcriptionally active chromatin (Fig. 2C), which was confirmed by *in vivo* transcription assays based on the incorporation of 5′-fluorouridine (FU) into nascent RNA, followed by immunofluorescence using an anti-BrdU antibody (Fig. 2C, lowest panels). Moreover, immunoelectron microscopy analyses using double immunogold labeling for 5′-FU incorporation sites and DKK-1 revealed the presence of DKK-1 in euchromatin but not in transcription-free nuclear domains such as heterochromatin or nuclear speckles visualized as interchromatin granule clusters (IGC) (Fig. 2D, above). Moreover, specific sites of colocation of nascent RNA and DKK-1 were found (Fig. 2D, below).

We examined whether ectopic DKK-1 could affect Wnt/β-catenin or non-canonical Wnt signaling in DLD-1 cells. DKK-1 did not affect the transcriptional activity of β-catenin (Supplementary Fig. S3A) or the expression of two Wnt/β-catenin targets, c-Myc and cyclin D1 (Supplementary Fig. S3B). Likewise, DKK-1 did not change the levels of total or phosphorylated JNK or of GSK-3β activity in the cytosol (Supplementary Fig. S3C and D). Taken together, these results indicate that the tumor suppressive action of DKK-1 in DLD-1 cells [8] in which the Wnt/β-catenin pathway is endogenously activated due to *APC* mutation is independent of Wnt.

### Nuclear DKK-1 in tumor cells associates with worse progression-free and overall survival in CRC patients

**Figure 3:**
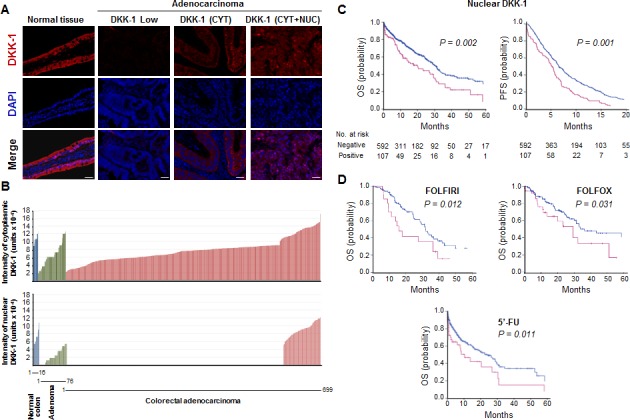
Nuclear DKK-1 expression decreases during CRC progression and associates with shorter patient PFS and OS A, immunofluorescence for DKK-1 and DNA (DAPI) in healthy colon tissue and in adenocarcinomas that express only cytosolic (CYT), cytosolic and nuclear (CYT+NUC) or no (negative) DKK-1 protein. Scale bars: 25 μm. B, quantification of DKK-1 expression in cytoplasm and nucleus of healthy colon tissue, adenomas and adenocarcinomas. The number of samples is indicated. C, and D, association of nuclear DKK-1 with worse PFS and OS (Kaplan-Meier curves) of CRC patients who received any systemic (C) or specific chemotherapy regimens (OS) (FOLFOX, FOLFIRI, 5′-FU) (D). Red curves: patients with nuclear DKK-1 expression; blue curves: absence of nuclear DKK-1 expression. Patients at risk for the specific event are detailed for each time point.

Next, we examined the expression of DKK-1 protein in adenomas and a cohort of 699 CRC patients with clinical follow-up (Supplementary Table S1). As in healthy individuals, DKK-1 protein was detected both in the nucleus and in the cytoplasm of normal enterocytes in adjacent non-tumoral colorectal mucosa; in contrast, three patterns of expression were found in tumors: absence of DKK-1, presence of only cytoplasmic DKK-1, and presence of DKK-1 in both cytoplasm and nucleus (Fig. 3A). Staining of complete sections of colorectal tumors showed that the pattern of DKK-1 protein expression was mostly homogeneous; no differences were found between tumor areas. The nuclear localization of DKK-1 protein was confirmed by using different antibodies (Supplementary Fig. S4). Data quantification across the series revealed absence of nuclear DKK-1 in 22% of adenomas and 75% of carcinomas, and a loss of DKK-1 expression during tumor progression that occurred earlier in the nucleus than in the cytoplasm (Fig. 3B). Remarkably, nuclear DKK-1 levels remained high in a subset (*n* = 103, 14.7%) of CRC. This prompted us to analyze whether the presence of nuclear DKK-1 within the primary tumor could affect clinical behavior. Nuclear DKK-1 expression was associated with decreased Progression-Free Survival (PFS) after chemotherapy administration (*P* = 0.001) and Overall Survival (OS) (*P* = 0.002) (Fig. 3C). The hazard ratio for death in patients with nuclear DKK-1 expressing tumors was 1.65 (IC 95%; 1.23-2.21). This remained true for patients who received 5′-FU (*n* = 399, *P* = 0.011), FOLFIRI (*n* = 95, *P* = 0.012) or FOLFOX (*n* = 205, *P* = 0.031) for both OS (Fig. 3D) and PFS (Supplementary Fig. S5). These findings suggested a role of nuclear DKK-1 in chemosensitivity and survival in CRC patients.

### DKK-1 regulates the expression of the cancer-related genes *ALDH1A1* and *REPS2*

**Figure 4:**
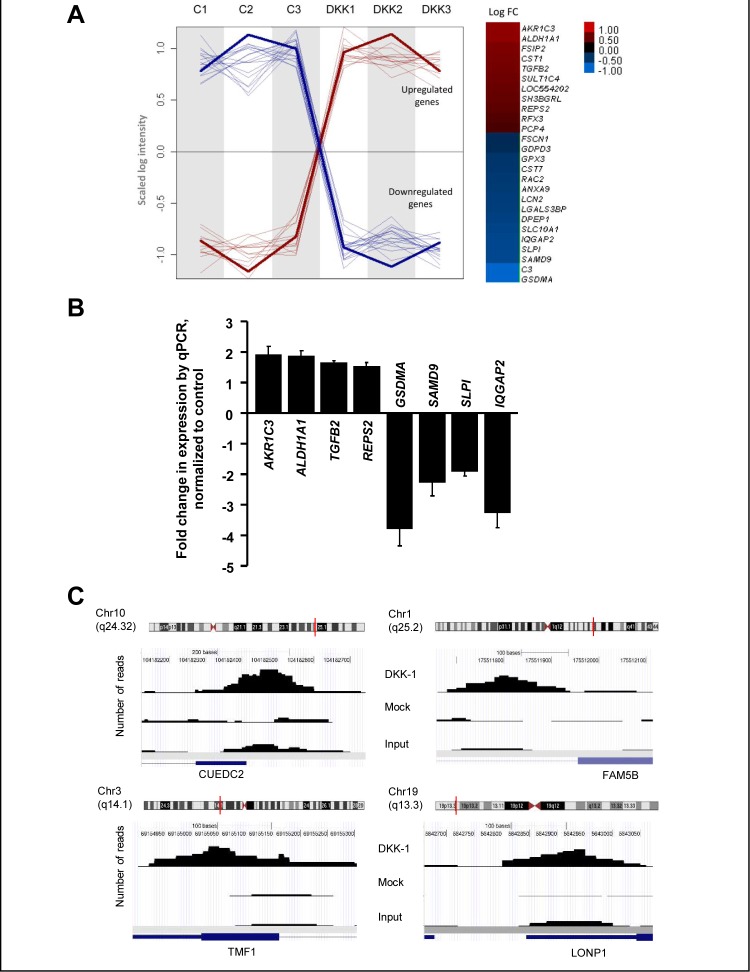
Effects of DKK-1 induction on gene expression in DLD-1 cells A, left, plot profile of the expression values along arrays for the probes selected as differentially expressed (P*adjust* 0.05) after DKK-1 induction. Signal intensities are scaled to the same range and the normalized log intensity values of the probes were centered to the median value of each probe set. A *loess* (locally weighted polynomial regression) fit line describes the overall profile of the up-regulated (*red*) and down-regulated (*blue*) genes. Right, log_2_ Fold-Change (FC) representation of the differentially expressed genes. The color intensity is proportional to the value (DKK-1 is not included). B, validation of DKK-1 target genes in DLD-1/DKK-1 cells. RNA levels were measured by qRT-PCR 24 h after DKK-1 induction by doxycyclin. Fold-change with respect to levels in DLD-1/Mock cells is represented. C, ChIP-seq analysis of DKK-1 in DLD-1 cells. Examples of the genome-wide mapping of DKK-1 binding sites. Significant DKK-1 peaks detected by Model-based Analysis of ChIP-Seq are represented in the samples analyzed. The corresponding Refseq gene is shown in blue. The vertical red line in the chromosome indicates the location of the peak.

The location of nuclear DKK-1 in a subset of sites of active transcription indicates that it participates in the regulation of specific target genes. To identify DKK-1 targets we used DLD-1/DKK-1 and DLD-1/Mock cells to perform: a) a comparative global transcriptomic analysis; and b) chromatin immunoprecipitation assays followed by ultradeep sequencing (ChIP-seq). We obtained 27 differentially expressed probes (Fig. 4A; the complete list of genes is deposited in the GEO database accession no. GSE35272). Among candidate target genes identified in the transcriptomic studies, aldehyde dehydrogenase 1A1 (*ALDH1A1),* aldo-keto reductase family 1 member C3 (*AKR1C3)* and Ral-binding protein 1-associated Eps domain-containing 2 (*REPS2)* were particularly interesting as they have been implicated in the detoxification of xenobiotics and chemotherapeutic agents [22-25] (Fig. 4B). Moreover, *ALDH1A1* has been proposed as a marker of normal and malignant stem cells in colon and other tissues, although there is no consensus on its association with prognosis or patient survival [26-28]. In the ChIP-seq, 200 binding sites (average peak width 150) with significant tag enrichment were identified throughout the genome (Fig. 4C). Up to 80 individual DKK-1-binding sites (40%) were associated with genes (GEO accession no. GSE31170), three of which were coincident with those found in the transcriptomic study (*REPS2, IQGAP2, LOC554202*).

**Figure 5:**
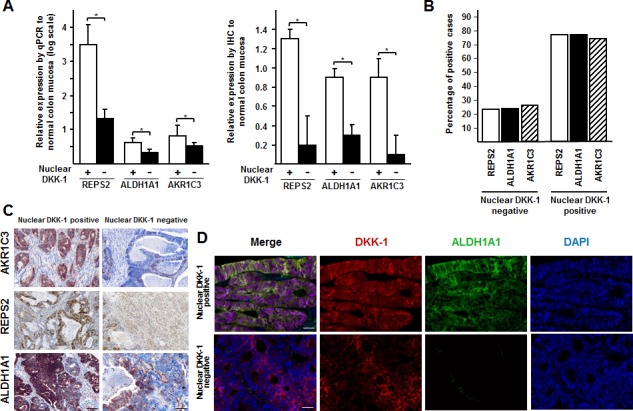
DKK-1 induces *ALDH1A1, REPS2* and *AKR1C3* genes involved in chemotherapy detoxification A, expression of *ALDH1A1, REPS2* and *AKR1C3*genes at the RNA (left, qRT-PCR) and protein (right, immunohistochemistry) level in primary (*n* = 12) and metastatic (*n* = 13) colon tumors expressing or not nuclear DKK-1 as compared to healthy (*n* = 10) colon mucosa (*, *P* < 0.05). B, association of nuclear DKK-1 with *ALDH1A1, REPS2* and *AKR1C3* expression in the series of 699 CRC patients. C, immunohistochemical analysis of *ALDH1A1, REPS2* and *AKR1C3* expression in representative nuclear DKK-1-positive (strong staining) and -negative (weak staining) tumors. Scale bars: 50 μm. D, immunohistochemical analysis of DKK-1 and ALDH1A1 protein co-expression in tumors. Scale bars: 25 μm.

Consistent with their regulation by DKK-1, the RNA and protein expression of *ALDH1A1, AKR1C3* and *REPS2* was higher in CRC tumors containing nuclear DKK-1 than in those that lacked it (Fig. 5A) and it was associated with the presence of nuclear DKK-1 (Fig. 5B and C). Only two (0.3%) cases expressed nuclear DKK-1 and none of the three target genes. Moreover, co-expression of nuclear DKK-1 and cytoplasmic ALDH1A1 was demonstrated in the same tumor cells (Fig. 5D).

### Concomitant expression of *ALDH1A1*, *REPS2*, and nuclear DKK-1 associates with chemoresistance in colorectal cancer

**Figure 6:**
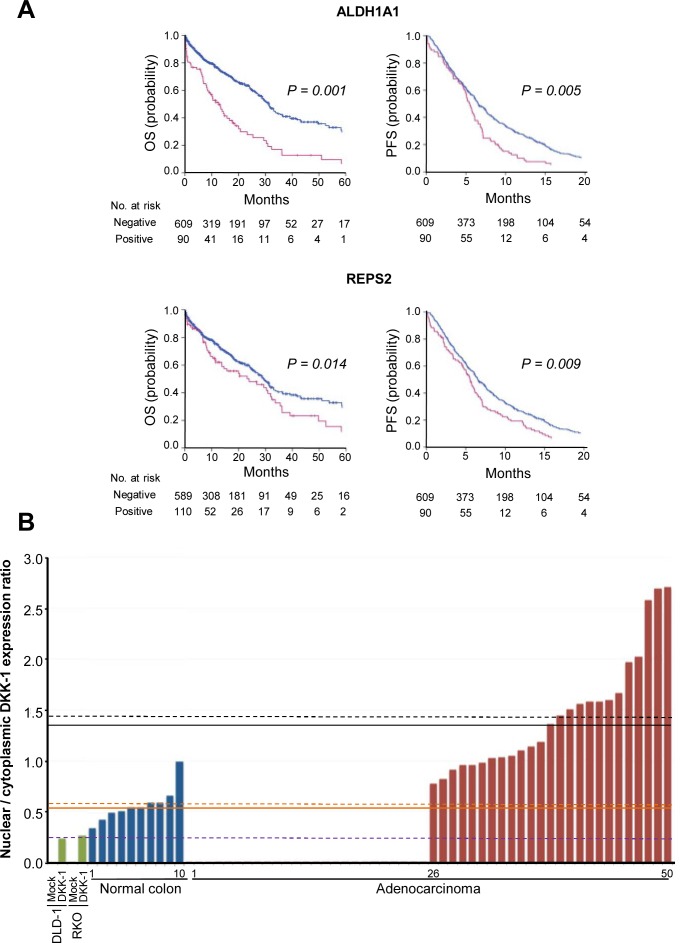
Expression of *ALDH1A1* and *REPS2* associates with decreased patient survival A, effect of *ALDH1A1* and *REPS2* genes on patient PFS and OS. Kaplan-Meier curves of patients whose primary tumors expressed (red) or not (blue) *ALDH1A1* or *REPS2* genes. B, nuclear-to-cytoplasmic ratio of DKK-1 expression in a subset of CRC (*n* = 50) and healthy colonic mucosa (*n* = 10) as compared with DLD-1 and RKO colon carcinoma cells lacking endogenous DKK-1 expression that were infected with lentiviruses encoding *DKK-1* (DKK-1) or no ectopic gene (Mock). Mean values for each group are indicated by dashed lines (black, CRC with nuclear DKK-1 expression; orange, healthy mucosa; purple, cell lines with DKK-1 expression) and median values by solid lines.

The relation of the three DKK-1 target genes with the benefit to chemotherapy was investigated in CRC patients. *ALDH1A1* and *REPS2* expression, but not that of *AKR1C3* (not shown), was associated with decreased OS (*P* = 0.001 and 0.014, respectively) and PFS (*P* = 0.005 and 0.009, respectively) (Fig. 6A and Table 1). Importantly, tumors with nuclear DKK-1 and co-expression of at least one of the three target genes (*n* = 101, 14.4%) showed a significant reduction in patient OS as compared to cases with absence of nuclear DKK-1 and expression of any of the three genes (*n* = 55, 7.9%) (*P* = 0.010). Notably, no effect of DKK-1 on sensitivity to oxaliplatin, irinotecan or 5′-FU was found *in vitro* (DLD-1 and RKO cells), possibly because ectopic *DKK-1* expression rendered a level of nuclear DKK-1 protein that was similar to that of the endogenous protein in SW480-ADH cells (7%, Fig. 2) but substantially lower than that found in tumors (Fig. 6B and Supplementary Fig. S6). In line with this, the expression level of ALDH1A1, REPS2, and AKR1C3 was lower in DLD-1/DKK-1 cells than in tumors with nuclear DKK-1 (Supplementary Fig. S7). This result suggests that extracellular signals, either paracrine soluble factors or matrix components, may contribute *in vivo*, but not *in vitro* where they are absent, to control the amount of nuclear DKK-1.

**Table 1:** Univariate and multivariate analysis for PFS and OS of nuclear DKK-1 expression in entire cohort of CRC patients

	Univariate PFS analysis					Multivariate PFS Cox analysis					Univariate OS analysis					Multivariate OS Cox analysis				
	HR	95% CI			Significance	HR	95% CI			Significance	HR	95% CI			Significance	HR	95% CI			Significance
		Lower		Upper			Lower		Upper			Lower		Upper			Lower		Upper	
Gender					0.527					-					0.223					-
Female	1.000										1.000									
Male	1.052	0.89	to	1.23		-	-		-		1.611	0.91	to	1.48		-	-		-	
ECOG					0.484					-					0.001					0.001
0-1	1.000										1.000					1.000				
2-3	1.067	0.88	to	1.28		-	-		-		2.995	2.31	to	3.88		2.850	2.21	to	3.85	
Number of metastatic sites					0.362					-					0.018					0.004
1-2	1.000										1.000					1.000				
>2	1.125	0.87	to	1.45		-	-		-		1.515	1.07	to	2.13		1.692	1.18	to	2.42	
Prior adjuvant chemotherapy					0.001					0.118					0.003					0.118
No	1.000					1.000					1.000					1.000				
Yes	0.629	0.52	to	0.75		0.613	0.51	to	0.74		0.642	0.48	to	0.85		0.783	0.57	to	1.06	
Treatment 1st line metastatic					0.937					-					0.001					0.142
5-FU	1.000										1.000					1.000				
Oxaliplatin	1.038	0.81	to	1.33		-	-		-		0.472	0.35	to	0.62		0.752	0.59	to	1.03	
Irinotecan	0.994	0.83	to	1.18		-	-		-		0.663	0.47	to	0.91		1.021	0.71	to	1.46	
Nuclear DKK-1					0.001					0.001					0.002					0.001
No	1.000					1.000					1.000					1.000				
Yes	1.622	1.31	to	2.01		1.699	1.36	to	2.11		1.602	1.19	to	2.15		1.654	1.22	to	2.24	

## DISCUSSION

Our results show for the first time that a proportion of DKK-1 is located in the nucleus of human enterocytes and malignant epithelial cells in CRC and regulates the expression of a number of genes including several that are relevant for cancer. The location of nuclear DKK-1 in a subset of sites of active transcription indicates that it is not an essential component of the chromatin or of the basal transcriptional machinery but, rather, that it participates in the transcription of specific target genes.

In recent years, the concept has emerged that proteins that exert a function in a given subcellular compartment may play additional roles in different locations [29]. Thus, cytoskeletal and even plasma membrane proteins have been found within the cell nucleus, in some cases being involved in gene transcription [30, 31]. The mechanism of DKK-1 translocation into the cell nucleus is unknown. Although the mature DKK-1 protein (37 kDa) does not have a conventional nuclear localization signal, it has been proposed that small molecules including proteins of molecular mass up to 40 kDa may enter the nucleus by passive diffusion [32] or be imported by interaction with other proteins that contain a functional nuclear localization signal [33].

The presence of a fraction of DKK-1 within the nucleus controlling gene expression might contribute to explain the recently proposed role of DKK-1 to inhibit colon cancer-initiating cell growth through mechanisms other than the blockade of the Wnt/β-catenin signaling pathway [34]. Curiously, the inhibition of cell proliferation observed in mouse intestinal crypts by forced expression of exogenous Dkk-1 has been interpreted as the requirement of Wnt/β-catenin signaling [35]. Likewise, increased proliferation of colon epithelial cells upon reduced expression of Dkk-1 has been associated with increased transcriptional activity of β-catenin [36]. Our data, however, provide an alternative explanation for the β-catenin-independent growth inhibitory effects of DKK-1 in DLD-1 colon carcinoma [8], H28 and MS-1 mesothelioma [7] and HeLa cervical cancer [37] cells.

Recently, it has been demonstrated that colorectal cancer cells can respond to exogenous and autocrine Wnt ligands that further activate the Wnt/β-catenin signaling pathway despite having mutations that stabilize β-catenin [38]. Therefore, the idea that extracellular Wnt inhibitors have novel functions based on the earlier assumption that mutations in APC or β-catenin would render cells completely independent of canonical Wnt/β-catenin signaling needs to be re-evaluated. Our data show that DKK-1 does not affect β-catenin/TCF transcriptional activity in *APC/KRAS* mutant DLD-1 cells (Supplementary Fig. S3A) and we propose that nuclear DKK-1 plays an important functional role in these cells, as well as *in vivo*. However, we cannot rule out effects of extracellular DKK-1 mediated through LRP5/6 binding at the plasma membrane.

The apparent paradoxical behavior of DKK-1 as a tumor suppressor and an inducer of chemoresistance may be explained by its distinct roles in the cytoplasm and nucleus and the non-synchronous loss of its expression in both cell compartments. The decrease in nuclear DKK-1 found at early stages may facilitate tumor progression based on the results of the ChIP analysis: nuclear DKK-1 regulates genes with antiproliferative action (i.e., *NRG1, NRG3, MAP2K3, PREX2, FAM5*B) and involved in apoptosis (i.e., *BCL2A1, DAPK1*) (GSE31170), which may add to the action of extranuclear DKK-1 inhibiting β-catenin target genes such as *c-MYC* or *CCND1*/cyclin D1. At later stages in human tumors, systemic therapy can cause the selection of tumor cells with high nuclear DKK-1 that by inducing genes such as *ALDH1A1* and *REPS2* may be responsible for chemoresistance. Thus, DKK-1 seems to have a dual action in colon cancer, that is analogous to that of transforming growth factor (TGF)-β [39, 40], a suppressive action at early stages, by inhibiting cell proliferation by both Wnt-dependent and -independent mechanisms, and a tumor promoting action at later stages of progression, by inducing chemoresistance.

The induction by DKK-1 of *ALDH1A1* and *REPS2* genes that are involved in cellular detoxification explains the co-expression of nuclear DKK-1 and these target genes in tumors of patients with poor outcome after chemotherapy. Thus, *ALDH1A1* and *REPS2* (mutated in 14/700, (2%) of colon tumors, and in 4/700, (0.57%) of colon tumors, respectively; Cosmic database) seem to be major mediators of nuclear DKK-1 effects. The precise mechanism by which DKK-1 induces *ALDH1A1* remains to be elucidated. In mouse osteosarcoma cells, Dkk-1 has been recently shown to activate ALDH1 via the non-canonical Jun-mediated Wnt pathway [41], which however is not affected in our human CRC cells. For REPS2, the coincidence of transcriptomic and ChIP-Seq analyses indicates a direct transcriptional regulation by DKK-1.

A number of recent studies propose an important role of ALDH1A1 in the response to chemotherapy of patients with colon cancer or other neoplasias. Thus, colon cancer stem cells secrete high levels of ALDH1A1 that cause extracellular detoxification of maphosphamide [23] and ALDH1A1 also confers resistance of COLO 205 cells to cyclophosphamide [42]. Consistently, ALDH1A1 expression in colorectal adenoma is associated with a higher risk of metachronous adenoma independent of adenoma size or histopathology [43]. CRC patients with circulating tumor cells expressing ALDH1, survivin and MRP5 have shorter PFS [44], and ALDH1A1 rs1342024 polymorphism associates with shorter time to tumor recurrence [26]. ALDH1A1 is also associated with worse PFS and OS in clear cell renal cell carcinoma [45] and breast cancer patients treated with neo-adjuvant chemotherapy [46]. Moreover, ALDH1A1 expression associates with chemoresistance of cultured and xenografted ovarian cancer and pancreatic adenocarcinoma cells [47, 48]. Likewise, gastric cancer cells with high level of ALDH1A1 expression are more resistant to 5-FU and *cis*-diamminedichloroplatinium than those cells with lower level [49].

Our group has previously reported that *DKK-1* gene expression is downregulated in colon cancer as compared to healthy adjacent tissue [50] and that this is partially due to promoter methylation of the gene in advanced stages of progression (Dukes' C and D) [8]. Those studies were performed assessing the relative amount of *DKK-1* RNA in healthy vs. tumor tissue. In view of our current work, it is not only the amount but also the location of DKK-1 that matters, so we suggest that future assessments of DKK-1 status, at least in CRC, must be performed by immunodetection procedures.

We previously established that the half-life of *DKK-1* RNA is 2.0 h in SW480-ADH cells [9] and it has been reported to be 2.3 h in HeLa cells [51] and 2.25 or 1 h in SW480 cells depending on the culture medium [52]. We are not aware of any study of DKK-1 protein stability and do not know whether nuclear and extranuclear DKK-1 protein differ in half-life.

In summary, we provide evidence for unpredicted, Wnt-independent gene regulatory activity of DKK-1 within the nucleus that is associated with chemoresistance and decreased survival in CRC patients. Our findings may help to clarify the role of DKK-1 as a tumor suppressor or metastasis promoter [21]. Discrepancies and conflicting data present in the literature may depend not only on the type of cancer but, at least in colorectal neoplasias, also on the location of DKK-1 protein and the stage of tumor progression: the presence of DKK-1 within the cell nucleus seems to be specifically responsible for drug resistance and so, the analysis of the subcellular distribution of DKK-1 is warranted. We propose nuclear DKK-1 as a biomarker for predicting benefit to systemic therapy in metastatic CRC.

## METHODS

### Cell culture

SW480-ADH, DLD-1 and RKO cells were cultured in DMEM supplemented with 10% fetal calf serum (FCS) (both from Invitrogen). Cell lines were originally obtained from the American Type Culture Collection (ATCC) and authenticated using the *GenePrint*® 10 System (Promega), which allows co-amplification and three-color detection of ten human *loci*: TH01, TPOX, vWA, Amelogenin, CSF1PO, D16S539, D7S820, D13S317, D21S11 and D5S818. Short Tandem Repeats profiles were sent for comparison against cell line datebases (ATCC, DSMZ). Last test was done on February 2014. Immortalized mouse neonatal hepatocytes were provided by Dr. A. Martínez (Instituto de Investigaciones Biomédicas, Madrid). For transfection we used the jetPEI reagent (PolyPlus Transfection). *Firefly* (Luc) and *Renilla reniformis* luciferase (Rluc) activities were measured separately using the Dual Luciferase kit (Promega). Luc activity was normalized to Rluc activity. All experiments were performed at least in triplicate. To study β-catenin transcriptional activity we used the TOPFlash and FOPFlash plasmids provided by Dr. H. Clevers (Utrecht, The Netherlands).

### Subcellular fractionation

Whole-cell extracts were obtained in RIPA buffer as described elsewhere [53], and cytosolic and nuclear fractions were obtained as reported [54]. Protein concentration was measured using the Bio-Rad DC protein assay kit.

### Patients

Formalin-fixed paraffin-embedded 3 μm tissue sections from human non-pathological colon (*n* = 16) and small intestine (*n* = 16), colorectal adenomas (*n* = 76) and primary tumors from metastatic colorectal (mCRC) patients (*n* = 699) were retrieved from Fundación Jiménez Díaz Biobank. Tumor specimens were retrospectively selected from consecutive mCRC patients (1998-2009), which had fulfilled the following criteria: adenocarcinoma, metastatic disease, no neoadjuvant therapy, available tissue and clinical follow up. Demographic data are shown in Supplementary Table S1. Clinical data and follow up were collected from medical clinical records by medical oncologists. The study was approved by the Ethics Committee of the institution (PIC 23/2012).

### Immunofluorescence, immunohistochemical and confocal microscopy analyses of human tissues and cultured cells

Staining of human tissues was performed as described [50] using anti-DKK-1 (Cell Signaling Technology, #4687; Santa Cruz Biotechnology, sc-14949), anti-ALDH1A1 (R&D Systems, MAB5869, clone 703410), anti-REPS2 (Novus Biologicals, NBP1-80891), anti-AKR1C3 (Abcam, ab170530), and anti-chromogranin A (Dako, M0869, clone DAK-A3) antibodies. DKK-1 expression was determined in primary tumors (diagnostic samples) before chemotherapy administration. Intensity for nuclear and cytoplasmic DKK-1 was scored by a computerized measurement using the Nuance FX Multispectral Imaging System (CRI Caliper Life Sciences) as described [55] by two researchers (FR and SZ). For immunofluorescence analyses, cells were rinsed once in PBS, fixed in 3.7% paraformaldehyde for 15 min at RT and rinsed once in 0.1 M glycine and twice in PBS. They were permeabilized in 0.5% Triton X-100 and then washed three times in PBS. The non-specific sites were blocked by incubation with PBS containing 1% goat serum for 30 min at RT. Next, cells were incubated with a rabbit or goat polyclonal antibodies against DKK-1 (Cell Signaling Technology, #4687; Santa Cruz Biotechnology, sc-14949), mouse monoclonal against V5-epitope (Invitrogen, R960-25) and either goat polyclonal antibodies to lamin B (Santa Cruz Biotechnology, sc-6216) or mouse monoclonal antibodies to fibrillarin (Abcam, ab4566, clone 38F3) or to TMG-cap which recognizes the 5′ cap structure of spliceosomal snRNAs (Oncogene Research Products, NA02, clone K121) diluted in PBS for 3 h at RT or overnight at 4°C. After four washes in PBS, cells were incubated with secondary antibodies for 45 min at RT, washed and mounted in VectaShield (Vector Laboratories). Confocal microscopy was performed with a LSM510 laser scanning microscope (Carl Zeiss) equipped with argon (488 nm), HeNe (543 nm) and HeNe (633 nm) ion lasers. All confocal scans were acquired with the LSM510 software using a Plan Apochromat 63x NA 1.4 objective (Carl Zeiss). For double or triple labeling experiments, images of the same confocal plane were sequentially recorded and pseudocolor images were generated and superimposed. TIFF images were further processed using Photoshop (CS3, Adobe Systems) for presentation.

### Western blotting

Western blotting analyses were performed as described [53]. Antibodies: DKK-1 (Santa Cruz Biotechnology, sc-14949; Cell Signaling Technology #4687), β-tubulin (Sigma-Aldrich, T4026), c-MYC (Santa Cruz Biotechnology, sc-40, clone 9E10), cyclin D1 (Santa Cruz Biotechnology sc-718), lamin B (Santa Cruz Biotechnology, sc-6216), V5 (Invitrogen, R960-25), β-actin (Cell Signaling Technology, #5125, clone 13E5), phospho-JNK (Cell Signaling Technology, #9251), JNK (Santa Cruz Biotechnology, sc-474), phospho-GS S641 (Cell Signaling Technology, #3891), GS (Cell Signaling Technology, #3886, clone 15B1). Specific DKK-1 antibody blocking peptide (Santa Cruz Biotechnology, Y-17P) was used for control purposes.

### *In situ* transcriptional activity

Active transcription sites were labelled by incorporation of 5′-fluorouridine (5′-FU, Sigma) into nascent RNA as described [56]. Briefly, cells were grown to subconfluence on glass coverslips. 5′-FU was added to the culture medium at 2 mM for 20 min. The cells were then fixed in 3.7% paraformaldehyde in HPEM buffer (HPEM 2x: 60 mM Hepes, 130 mM Pipes, 20 mM EGTA, and 4 mM MgCl_2_.6H_2_O) containing 0.5% Triton X-100 for 10 min. The incorporation of 5′-FU into nascent RNA was detected by incubation for 1 h at 37°C with a mouse monoclonal antibody against halogenated UTP (anti-BrdU, Sigma, clone BU-33), diluted 1:50 in PBS. The samples were then washed in 0.01 % Tween 20 in PBS, incubated for 45 min with anti-mouse FITC-conjugated secondary antibody (Jackson ImmunoResearch), and mounted with the anti-fading medium Vectashield (Vector Laboratories).

### Immunoelectron microscopy

For double immunogold electron microscopy detection of nascent RNA and DKK-1, SW480-ADH cells were cultured in the presence of 5′-FU to a final concentration of 2 mM for 20 min. Cells were fixed with 4% paraformaldehyde and 0.1% glutaraldehyde in 0.1 M cacodilate buffer for 30 min at RT. After fixation, cells were scrapped off, transferred to an eppendorf tube, and centrifuged for 10 min at 12,000 rpm in a minifuge. Cell pellets were washed with 0.1 M cacodylate buffer, dehydrated in increasing concentrations of methanol at −20°C, embedded in Lowicryl K4M at −20°C, and polymerized by ultraviolet irradiation. Ultrathin sections of 60 nm thickness were obtained by using an ultramicrotome (Ultracut UCT, Leica, Germany), mounted on nickel grids, and sequentially incubated with 0.1 M glycine in PBS for 15 min and 5% BSA in PBS for 30 min, and the primary antibodies diluted in 50 mM Tris HCl (pH 7.6) containing 1% BSA and 0.1 M glycine. The incorporation of 5′-FU into nascent RNA was detected with the mouse monoclonal anti-BrdU antibody; sections were incubated for 2 h at 37°C. After labeling for 5′-FU incorporation, ultrathin sections were incubated with a rabbit polyclonal anti-DKK1 antibody (Cell Signaling Technology, #4687) overnight at RT. Subsequently, ultrathin sections were then incubated with secondary antibodies, goat anti-rabbit or anti-mouse IgG, coupled to 10 nm or 15 nm gold particles, respectively (BioCell; diluted 1:50 in PBS containing 1% BSA). Following immunogold labeling, the grids were stained with uranyl acetate and examined under a Phillips EM208 electron microscope operated at 60 kV. As controls, ultrathin sections were treated as described above but omitting primary antibodies.

### ChIP-sequencing assay

Chromatin immunoprecipitation (ChIP) experiments were performed basically as described [57] using a DKK-1 antibody from Cell Signaling Technology (#4687). ChIP samples were processed into sequencing libraries with a ChIP-Seq sample preparation kit (Illumina) in accordance with the manufacturer's instructions. Briefly, each sample was electrophoresed on agarose gel and a fraction of 100-200 bp was taken. Extracted DNA was processed through subsequent enzymatic treatments of end-repair, dA-tailing, and ligation to adapters as in Illumina's “ChIP Sequencing Sample Prep Guide” (part # 11257047 Rev. A), with the exception that gel extraction was replaced with Agencourt AMPure XP (Beckman Coulter) bead purification. Each adapter-ligated library was PCR amplified with Illumina PE primers for 15 cycles. Independent aliquots were done with Phusion High-Fidelity DNA pol (Finnzymes Reagents) and Accuprime GC Rich pol (Invitrogen), and pooled after amplification. The resulting purified DNA libraries were applied to an Illumina flow cell for cluster generation and sequenced on the Genome Analyzer IIx (GAIIx; Illumina) following manufacturer's protocols.

Unfiltered 40 bp sequence reads were aligned against the human reference genome hg18 (NCBI build 36.1, March 2006) using Illumina's ELANDv2 algorithm on its “eland_extended” mode from within CASAVA-1.7 package. Raw sequences were defined as reads passing purity filter before genome alignment. Only the reads with a unique alignment in the reference genome (“s_N_sorted.txt” files) were used for the peak detection, which was performed with MACS (Model-based Analysis of ChIP-Seq). Peaks were called selecting default parameters for *P* value cut-off (default 1e-5) and modelfold (10, 30) for high-confidence fold-enrichment, where genomic DNA of mock and input samples were used as negative controls. The experimental settings, sequences and analysis protocols of the ChIP-seq experiment are deposited in GEO accession no. GSE31170.

### Gene expression analysis

Microarray analyses were performed using RNAs obtained from three independent dishes of DLD-1/Mock and DLD-1/DKK-1 cells that were treated with doxycyclin (10 μg/ml) for 24 h. Total cellular RNA was isolated using the RNeasy kit (Qiagen) according to supplier's specifications. The quantity and quality of the total RNAs obtained were determined using 6000 Nano Chips (Agilent Technologies). Total RNA samples were then processed for hybridization on Gene Chip Human Gene 1.0 ST Array (Affymetrix) using standard Affymetrix protocols at the Genomics and Proteomics Facility of Centro de Investigación del Cáncer (Salamanca, Spain).

Analysis for differential expression was performed using the R platform for statistical analysis (R Foundation for Statistical Computing, Vienna) and several packages from the Bioconductor project (http://www.bioconductor.org/). The raw data were imported into R and preprocessed using the *affy* package and the robust multichip average method. To identify differentially expressed genes, we used the *limma* package. Correction for multiple testing was accomplished by controlling the FDR using published methods [58]. The experimental settings and raw data obtained in this experiment are deposited in GEO accession no. GSE35272.

### Statistical analysis

Statistical analyses were conducted using SPSS v13.0 (SPSS Inc, Chicago, IL). All reported P values are two-sided. Prognostic and predictive effects were assessed using as clinical endpoints progression-free survival (PFS) and overall survival (OS) from metastatic event. PFS was calculated from the date of diagnosis of metastatic disease to the date when progression was confirmed. OS from metastatic event was determined as the time elapsed from the date of metastasis diagnosis to the date of death from any cause or the date of last follow-up. The prognostic analysis considered the influence of factors in all patients using simple Cox proportional hazards modeling on PFS and OS, and in multivariable models adjusting for other prognostic factors in this population. Kaplan-Meier curves were also plotted. For the predictive analysis, Cox proportional hazards models were used to estimate hazard ratios (HRs) with 95% CIs for metastatic treatment received stratified by factor status. This work was performed in accordance with Reporting Recommendations for Tumor Marker Prognostic Studies (REMARK) guidelines [59]. *In vitro* results were expressed as mean ± SD unless otherwise specified. ANOVA test was used for analysis of DKK-1 and target genes in human samples.

## SUPPLEMENTARY MATERIAL, FIGURES AND TABLE


