# Supplementary material for: Nuclear DICKKOPF-1 as a biomarker of chemoresistance and poor clinical outcome in colorectal cancer
Source: Oncotarget. 2015 Feb 28;6(8):5903–17. doi: 10.18632/oncotarget.3464 (PMC4467410; doi:10.18632/oncotarget.3464)
Supplement: Supplementary file 1 [file oncotarget-06-5903-s001.pdf]

Nuclear DICKKOPF-1 as a biomarker of chemoresistance and poor clinical outcome in colorectal cancer

Supplementary Material

Identification of nuclear DKK-1 by liquid chromatography-Mass spectrometric analysis

We performed a proteomic analysis of the electrophoretic band obtained after immunoprecipitation of nuclear extracts from SW480-ADH cells expressing an exogenous V5-tagged DKK-1 construct. The SDS-PAGE gel bands were cut and digested using modified porcine trypsin (Promega). Digests were analyzed on an Esquire Ultra IT mass spectrometer (Bruker), coupled to a nano-HPLC system (Proxeon). Peptide mixtures were initially concentrated on a 300 mm id, 1 mm PepMap nanotrapping column and subsequently loaded onto a 75 mm id, 15 cm PepMap nanoseparation column (LC Packings). An acetonitrile gradient (0-40% ACN/0.1% formic acid in water, in 45 min, flow rate ca. 300 nL/min) was used to elute the peptides through a PicoTip emitter nanospray needle (NewObjective) onto the nanospray ionization source of the IT mass spectrometer. MS/MS fragmentation (1.9 s, 100–2800 m/z) of two of the most intense ions was carried out, as detected from a 1.2 s MS survey scan (310–1500 m/z), using a dynamic exclusion time of 1.2 min for precursor selection and excluding single-charged ions. An automated optimization of MS/MS fragmentation amplitude, beginning at 0.60 V was used. Proteins were identified using Mascot (Matrix Science) to search the Swiss-Prot 57.9 database. MS/MS spectra were searched with a precursor mass tolerance of 0.4 Da, fragment tolerance of 0.7 Da, trypsin specificity with a maximum of 1 missed cleavage, cysteine carbamidomethylation set as fixed modification and methionine oxidation as variable modifications. Positive identification criterion was set as an individual Mascot score for each peptide MS/MS spectrum higher than the corresponding identity threshold score.

Quantitative RT-PCR (qRT-PCR)

Total RNA was extracted from cells with the RNeasy mini kit (Qiagen). Complementary DNA was synthesized from 0.5 μg of total RNA using the ImProm-II Reverse Transcription System (Promega). qPCR was performed using the 7500 StepOne Plus Real-Time PCR System and the Taqman Gene Expression Master Mix (Applied Biosystems) and a C1000™-CFX384 Thermal Cycler Real-Time System apparatus (Bio-Rad). Thermal cycling was initiated with a denaturation step at 95ºC for 10 min and consisted of 40 cycles (denaturation at 95ºC for 10 s, annealing and elongation at 60ºC for 60 s). Taqman probes (Applied Biosystems) used: AKR1C3 (Hs00366267_m1), ALDH1A1 (Hs00946916_m1), TGFB2 (Hs00234244_m1), REPS2 (Hs00190932_m1), GSDMA (Hs00937853_m1), SAMD9 (Hs_00415836_m1), SLPI (Hs00268204_m1), and IQGAP2 (Hs00183606_m1). The expression levels were normalized to the levels of ribosomal protein control RPLPO (Taqman probe set 4310879E). All experiments were performed in triplicate. Human samples: RNAs were purified from formalin-fixed paraffin-embedded samples human non-pathological colon (n = 10), and from primary tumors (n = 12) and liver metastsis (n = 13) of mCRC patients.

Construction of the *DKK-1* lentiviral vector and production of lentiviral particles

The Tet-On inducible lentiviral vector was based on the pRRL-cPPT-hPGK-mcs-WPRE with the reporter gene MuSEAP encoded under the control of the hPGK promoter (kindly provided by Dr. O. Danos, University College, London). To introduce the sequence of IRES-eGFP the MuSEAP was excised using MluI/EcoRV enzymes. The IRES-eGFP sequence was amplified from an intermediate vector, and restriction sites were introduced by PCR (forward 5’ acgcacgcgtgcccctctccctccc 3’; reverse 5’ acgcgatatctcgagtgcggccgcttta 3’), and then inserted into MluI/EcoRV sites. For the hDKK1-IRES-eGFP the cloning was done in two steps: first we introduced the hDKK1 sequence from the pCDNA3.1/V5HisA plasmid, into an

intermediate vector pENTR1A-IRES-eGFP, and restriction sites SalI/BamHI as well as a stop codon after the V5 sequence, were introduced by PCR (forward 5´ acgcgtcgacatgatggctctgggcgcagc 3'; reverse 5´acgcggatccttacgtagaatcgagaccga 3'). Secondly, and using the same strategy used for IRES-eGFP, the full sequence of hDKK-1-V5-pIRES-eGFP was amplified and restriction sites introduced by PCR (forward 5´acgcacgcgtatgatggctctgggcgc 3'; reverse 5´acgcgatatctcgagtgcggccgcttta 3'), and inserted into MluI/EcoRV sites. Lentiviruses were produced as previously described [1] For transduction of target cells, lentiviruses at a multiplicity of infection of 10-60 were added to the culture medium in the presence of 8 µg/ml polybrene (Sigma-Aldrich) for 8 h, typically yielding more than 95% transduced (GFP-positive) cells. For DKK-1 expression, transduced cells were cultured in the presence of doxycyclin (10 µg/ml).

Supplementary references

1. Punzón I, Criado LM, Serrano A, Serrano F, Bernad A. Highly efficient lentiviral-mediated human cytokine transgenesis on the NOD/scid background. Blood.2004; 103:580-582.

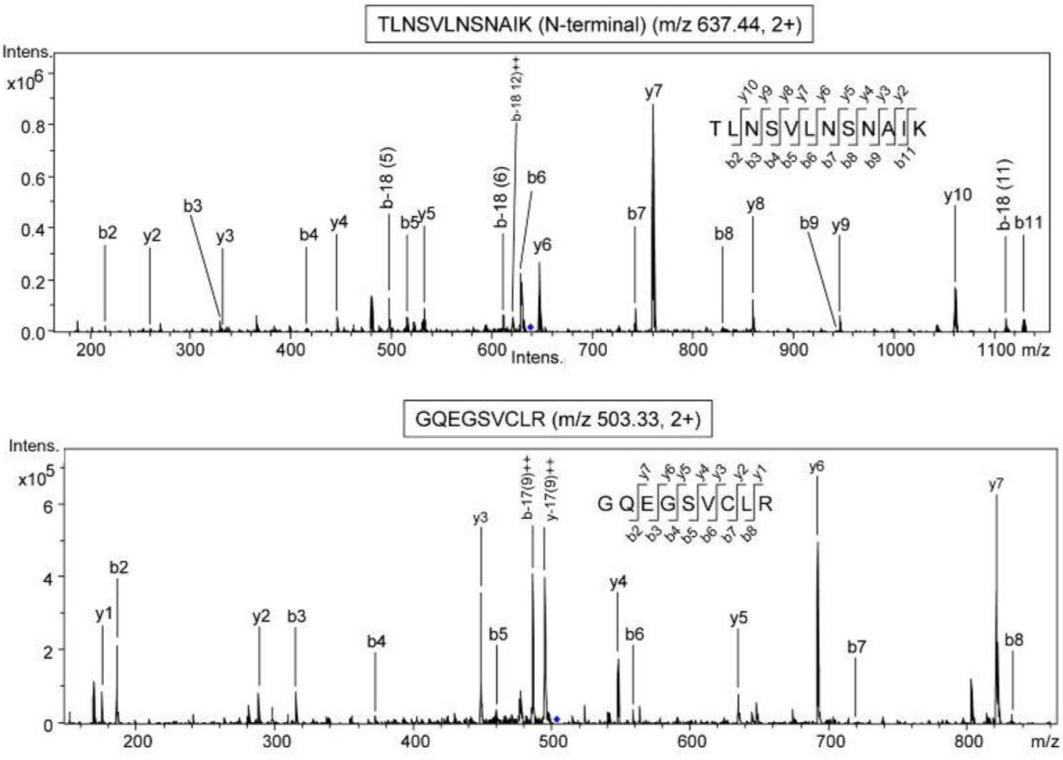

Supplementary Figure S1: Identification of the nuclear form of DKK-1 by mass spectrometry analysis. Annotated fragmentation mass spectra matching to the sequences of peptides spanning positions 32-43 (upper panel) and 183-191 of DKK-1_HUMAN sequence (lower panel). The sequence identified in upper panel corresponds to the N-terminal of the mature protein chain.

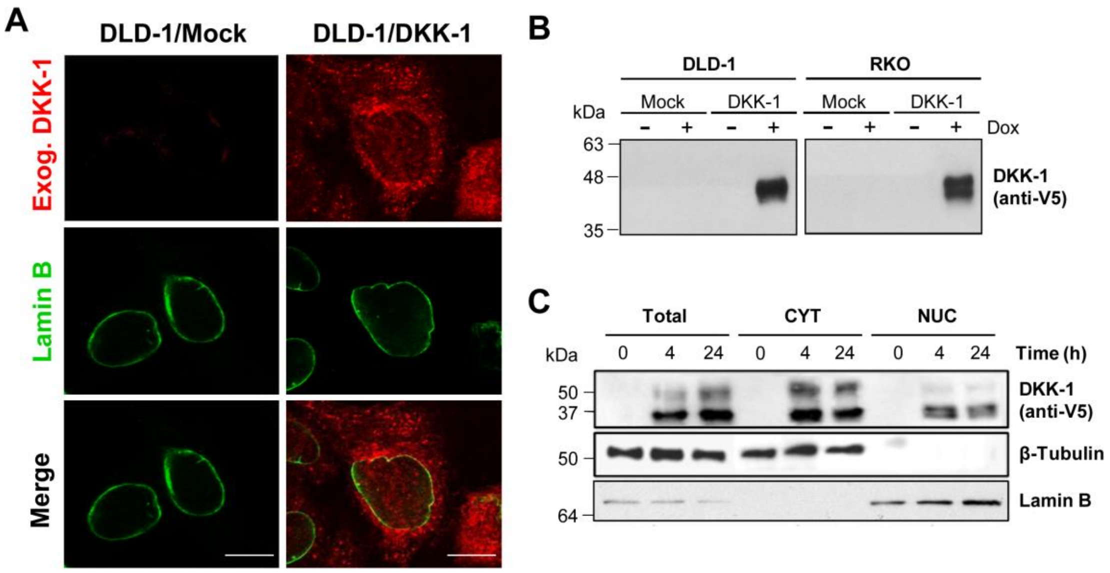

Supplementary Figure S2: Inducible ectopic DKK-1 expressed in DLD-1 cells is partially nuclear. A, representative images of DLD-1/Mock and DLD-1/DKK-1 cells after 24 h doxycyclin treatment showing cytoplasmic and nuclear DKK-1 immunofluorescence in the latter. Exogenous DKK-1 was detected with an anti-V5 antibody (Invitrogen, R960-25). Scale bars: 10 µm. B, expression of DKK-1 protein in the conditioned medium of DLD-1/Mock, DLD-1/DKK-1, RKO/Mock, and RKO/DKK-1 cells after 24 h doxycyclin treatment (10 µg/ml) by western blot analysis. C, expression of DKK-1 protein in total, cytoplasmic (CYT) and nuclear (NUC) fractions of DLD-1/DKK-1 cells at different times after doxycyclin treatment by western blot analysis. Lamin B and β-tubulin were used as nuclear and cytoplasmic markers, respectively. Nuclear fractions were concentrated ten times.

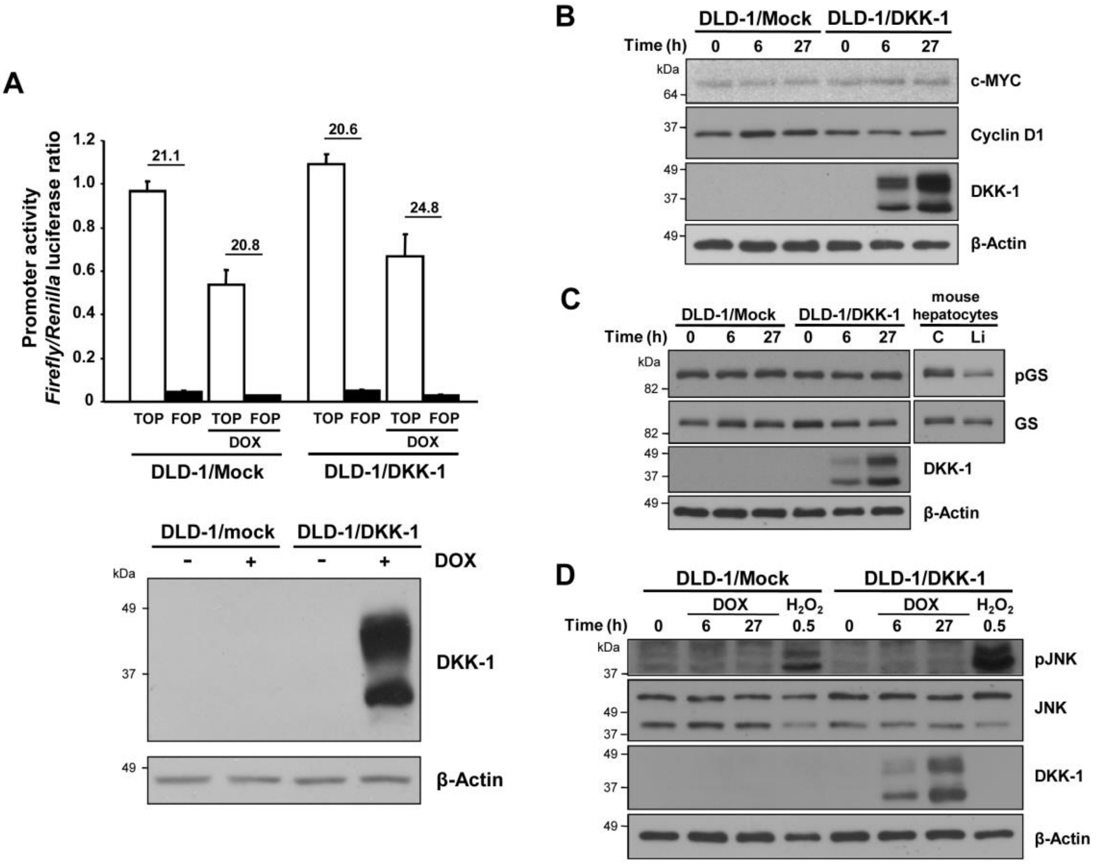

Supplementary Figure S3: DKK-1 expression did not affect canonical/β-catenin transcriptional activity or non-canonical Wnt signaling in DLD-1 cells. A, upper panel, β-catenin transcriptional activity in DLD-1 cells transfected with either TOPflash or FOPflash reporters and treated with doxycyclin (10 μg/ml, 48 h) as indicated. Lower panel, expression of DKK-1 protein analysed by western blot. β-Actin was used as loading control. B, expression of β-catenin targets c-Myc and cyclin D1 proteins in DLD-1/Mock and DLD-1/DKK-1 cells by western blot analysis. C, GSK-3β activity in DLD-1/Mock and DLD-1/DKK-1 cells. Levels of total and phospho-Ser^{641}-glycogen synthase (GS) measured by western blot analysis. Extracts from immortalized mouse neonatal hepatocytes treated with the GSK-3β inhibitor LiCl were used as control. D, levels of total and phosphorylated JNK in DLD-1/Mock and DLD-1/DKK-1 cells by western blot analysis.

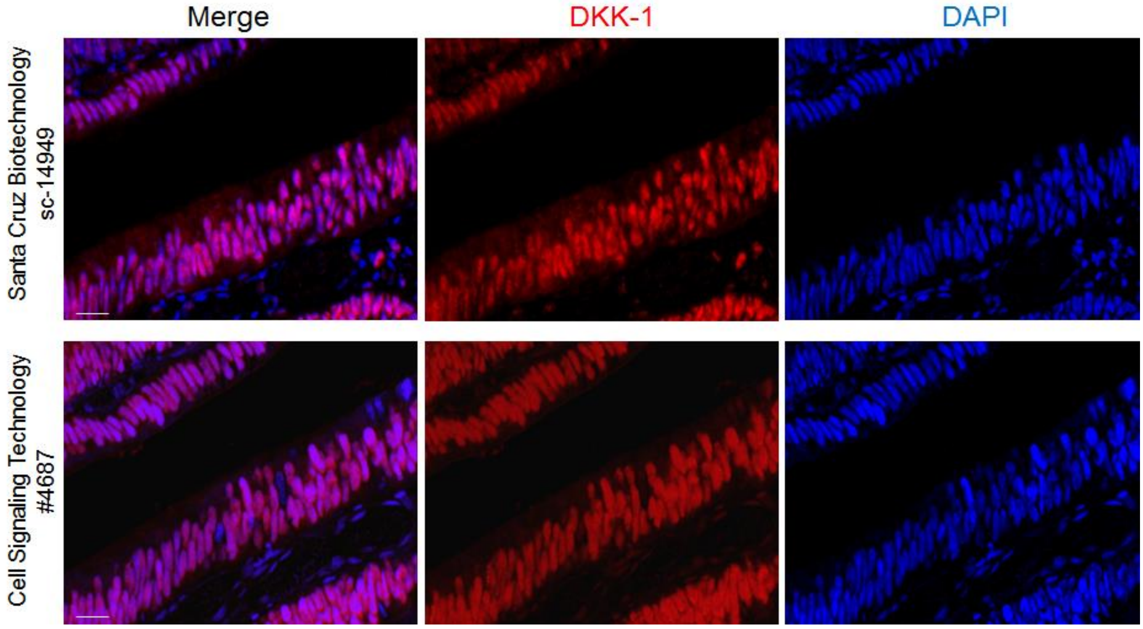

Supplementary Figure S4: Immunofluorescence analysis of DKK-1 expression in a well-differentiated colon carcinoma using two different commercial antibodies. The pattern of DKK-1 expression, nuclear and cytosolic, is the same for both antibodies. Scale bars: 10 μm.

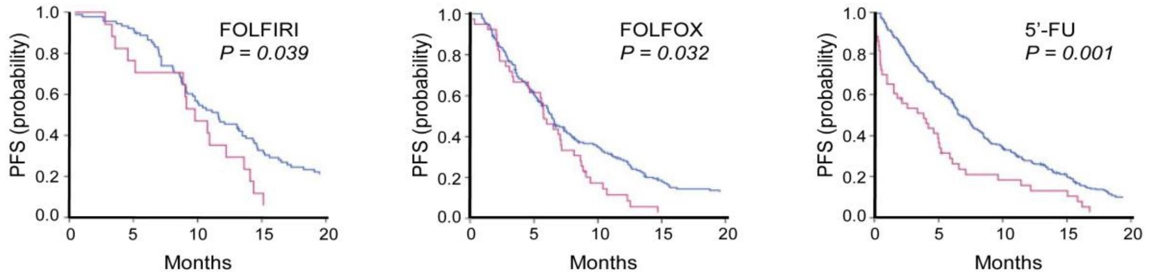

Supplementary Figure S5: Association of nuclear DKK-1 with worse PFS (Kaplan-Meier curves) of CRC patients who received specific chemotherapy regimens (FOLFOX, FOLFIRI, 5'-FU). Red curves: patients with nuclear DKK-1 expression; blue curves: absence of nuclear DKK-1 expression.

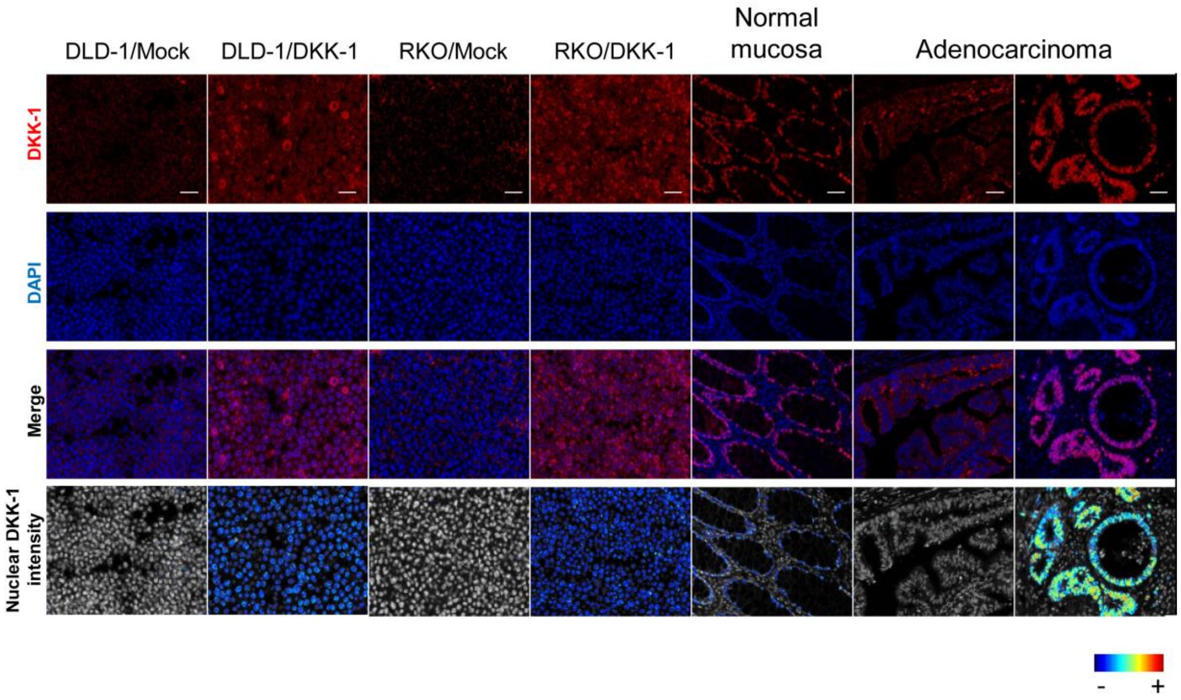

Supplementary Figure S6: Immunofluorescence analysis of DKK-1 expression in DLD-1/Mock, DLD-1/DKK-1 RKO/Mock and RKO/DKK-1 cells and, for comparison, in healthy colon mucosa and two CRC tumors expressing cytoplasmic DKK-1 and either absence or intense nuclear DKK-1 expression. Nuclear DKK-1 intensity was quantified by analysis of expression in nuclear masks. Scale bars: 25 μm.

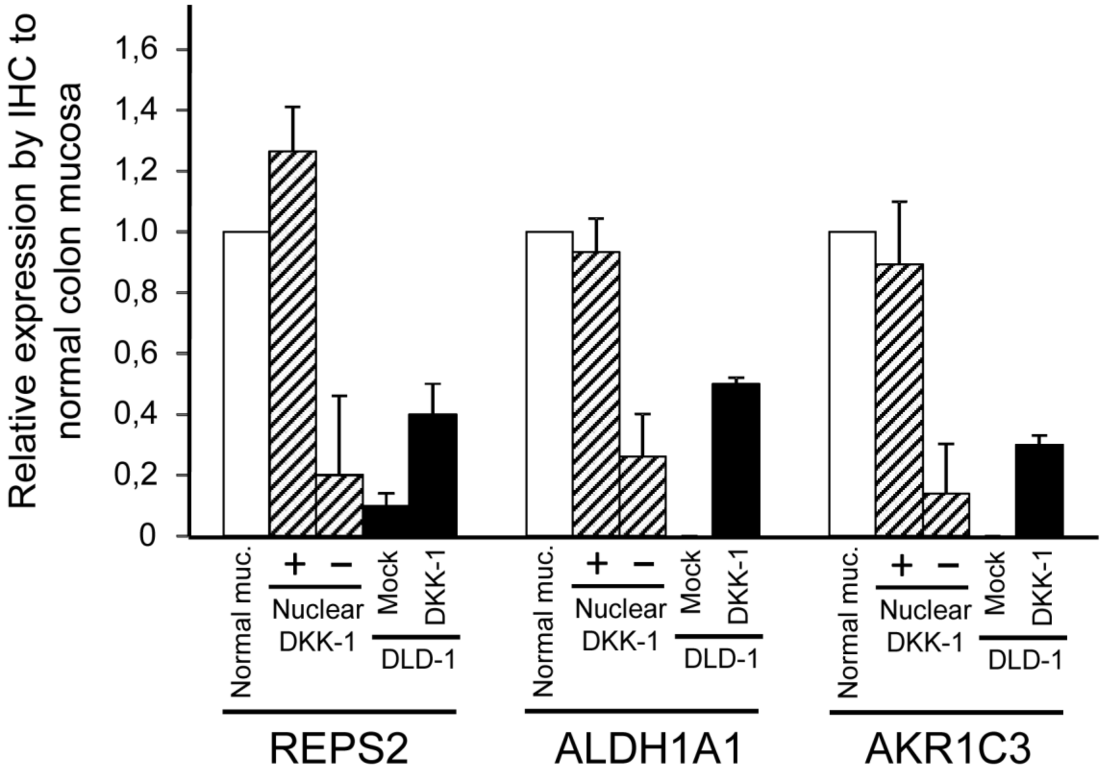

Supplementary Figure S7: Immunohistochemistry analysis of the expression of ALDH1A1, REPS2 and AKR1C3 proteins in human colon tumors expressing or not nuclear DKK-1, and in DLD-1/Mock and DLD-1/DKK-1 cells, as compared with colon healthy mucosa.

Supplementary Table 1: Demographic data of entire cohort of mCRC patients and DKK-1 expression

	Nuclear DKK-1 expression				
	Negative		Positive		Significance
	N	%	N	%	
Age, years					
Median	68		68		
Range	24-88		33-89		
Gender					0.031
Male	375	62.9	52	50.5	
Female	221	37.1	51	49.5	
ECOG					0.643
0-1	463	77.7	80	77.7	
2-3	133	22.3	23	22.3	
Number of metastatic sites					0.801
1-2	536	89.9	94	91.2	
≥2	60	10.1	9	8.8	
Liver metastases only					0.038
No	356	59.7	71	68.9	
Yes	240	40.3	32	31.1	
Prior adjuvant chemotherapy					0.689
No	442	74.2	81	78.6	
Yes	154	25.8	22	21.4	
Treatment 1st line metastatic					0.811
5-FU	325	54.5	56	54.4	
Oxaliplatin	174	29.2	31	30.1	
Irinotecan	79	13.3	16	15.5	
No therapy	18	3.0	0	0.0	
